# ACOEC-FD: Ant Colony Optimization for Learning Brain Effective Connectivity Networks From Functional MRI and Diffusion Tensor Imaging

**DOI:** 10.3389/fnins.2019.01290

**Published:** 2019-12-12

**Authors:** Junzhong Ji, Jinduo Liu, Aixiao Zou, Aidong Zhang

**Affiliations:** ^1^Beijing Key Laboratory of Multimedia and Intelligent Software Technology, Beijing Artificial Intelligence Institute, Faculty of Information Technology, Beijing University of Technology, Beijing, China; ^2^Department of Computer Science, University of Virginia, Charlottesville, VA, United States

**Keywords:** functional magnetic resonance imaging, diffusion tensor imaging, brain effective connectivity networks, anatomical constraint information, ant colony optimization

## Abstract

Identifying brain effective connectivity (EC) networks from neuroimaging data has become an effective tool that can evaluate normal brain functions and the injuries associated with neurodegenerative diseases. So far, there are many methods used to identify EC networks. However, most of the research currently focus on learning EC networks from single modal imaging data such as functional magnetic resonance imaging (fMRI) data. This paper proposes a new method, called ACOEC-FD, to learn EC networks from fMRI and diffusion tensor imaging (DTI) using ant colony optimization (ACO). First, ACOEC-FD uses DTI data to acquire some positively correlated relations among regions of interest (ROI), and takes them as anatomical constraint information to effectively restrict the search space of candidate arcs in an EC network. ACOEC-FD then achieves multi-modal imaging data integration by incorporating anatomical constraint information into the heuristic function of probabilistic transition rules to effectively encourage ants more likely to search for connections between structurally connected regions. Through simulation studies on generated datasets and real fMRI-DTI datasets, we demonstrate that the proposed approach results in improved inference results on EC compared to some methods that only used fMRI data.

## 1. Introduction

As an important method in brain science, brain imaging reveals the anatomic structure and function of a brain through images and imaging techniques such as functional MRI (fMRI), electroencephalography, magnetoencephalography, structural MRI and diffusion tensor imaging (DTI), and provides a powerful technical tool to understand the working mechanisms of the brain. Recently, the imaging data is often used to study connectivity in the human brain, that is, how brain regions interact with each other within networks to understand brain functioning and to handle cognitive processes (Friston, 2011). In particular, the resting-state fMRI data has been used to learn brain effective connectivity (EC) networks, which has aroused great interests among researchers. Different from the functional connectivity (FC) network, which is an undirected graph, an EC network is a directed graph where a node represents a brain region and a directed edge characterizes a causal effect of interval neural activity in the brain. By identifying and distinguishing brain EC differences between normal and abnormal subjects, people can understand the roles that connectivity patterns and their disruption play in mental health disorders and brain diseases, and can evaluate each abnormal brain EC and its relationship with injuries of neurodegenerative diseases, such as epilepsy, Alzheimer's disease (AD), schizophrenia, and autism, etc. Therefore, learning the brain EC from fMRI data can help elucidate the pathogenesis of cerebral diseases, which plays an important role in performing an early diagnosis of brain diseases and pathological studies.

Within the past decade, many computational methods and mathematical models have been proposed to identify EC involved in the human brain (Patel et al., 2006; Shimizu et al., 2006; Rykhlevskaia et al., 2008; Stephan et al., 2009; Dauwels et al., 2010; Ramsey et al., 2010; Seth, 2011; Smith et al., 2011; Sui et al., 2012; Wu et al., 2013; Zhu et al., 2013; Ide et al., 2014; Mumford and Ramsey, 2014; Zhou et al., 2015; Ji et al., 2016; Liu et al., 2016; Dang et al., 2017; Havlicek et al., 2017; Xu et al., 2017; Lennartz et al., 2018; Karwowski et al., 2019). These studies can be roughly divided into two categories, one is the model-driven approach while the other is the data-driven approach. The model-driven approaches usually require the prior models or hypothesis to conduct a valid connectivity analysis, which does not perform well for those situations where prior knowledge is insufficient (Wu et al., 2013). The data-driven approaches can directly extract causal interactions from fMRI data without any prior knowledge, and gradually become the mainstream method in identifying EC (Patel et al., 2006; Shimizu et al., 2006; Dauwels et al., 2010; Ramsey et al., 2010; Seth, 2011; Xu et al., 2017). Common methods belonging to the second category include: the Linear non-Gaussian acyclic model (LiNGAM) algorithm (Shimizu et al., 2006), The Granger causality (GC) algorithm (Seth, 2011), the Generalized synchronization (GS) algorithm (Dauwels et al., 2010), Patel's condition dependence measurement (Patel) algorithm (Patel et al., 2006), the Greedy equivalence search (GES) algorithm (Ramsey et al., 2010), and the Prediction Correlation (P-corr) (Xu et al., 2017) algorithm, etc. Though these methods have their own advantages in some ways, they have a common limitation on the direction estimation of EC. Recently, a data-driven approach based on Bayesian networks (BNs) has been greatly developed and has become an emerging approach for learning the brain EC (Ide et al., 2014; Mumford and Ramsey, 2014; Zhou et al., 2015). The main reason is that BN methods can accurately infer the functional connectivity between brain regions (Smith et al., 2011). However, they do not perform well on inferring causal directions. To overcome this problem, Ji et al. successively developed two swarm intelligent algorithms called AIAEC (Sui et al., 2012) and ACOEC (Liu et al., 2016) in 2016, which respectively use an artificial immune algorithm and an ant colony algorithm to infer EC between different brain regions. By means of two randomly global searching mechanisms in the candidate solution space, both AIAEC and ACOEC obtain higher accuracy on identifying the directions of EC compared with other methods. In particular, ACOEC not only has the same excellent identification ability on connections and directions of EC networks as that of AIAEC, but can also get the strengths of these connections, thus it is a more promising method of studying EC.

In recent years, multimodal analysis from multiple imaging data provides new insights for the progress of learning EC studies. This is because many studies have produced evidence, that FC based on fMRI is positively correlated with structural connectivity (SC) between brain regions based on DTI in the brain network (Rykhlevskaia et al., 2008; Sui et al., 2012; Zhu et al., 2013). Obviously, a multimodal analysis could provide a more reliable basis than a single modality analysis to differentiate brain patterns under various conditions such as normal, diseased, or aging (Dang et al., 2017). Up to now, there are some fusion methodologies for combining DTI and fMRI data (Zhu et al., 2013), for instance, a few studies have fused FC with axonal connectivity (AC). However, there are only two tentative studies (Stephan et al., 2009; Dang et al., 2017) that fuse EC with AC. Enno et al. used diffusion weighted imaging and probabilistic tractography to specify anatomically informed priors for dynamic causal models (DCMs) of fMRI data. More specially, the anatomical likelihood of a given connection was used to inform the prior variance of the corresponding coupling parameter in the DCM (Stephan et al., 2009). Dang et al. proposed a unified probabilistic framework that combines information from both DTI and fMRI data to learn EC using dynamic Bayesian networks, where a novel anatomically-informed score that simultaneously evaluates the fitness of a given connectivity structure to both sources was given (Dang et al., 2017). This type of research focuses on DCM-based methods using DTI and fMRI data. Therefore, how to further explore other novel fusing methods to identify EC from fMRI and DTI data is still an open and challenging research topic.

In this paper, a new method employing Ant Colony Optimization to learn EC from fMRI and DTI data is presented, named ACOEC-FD. First, ACOEC-FD uses DTI data to acquire some positively correlated relations among regions of interest (ROIs), and takes them as anatomical constraint information to effectively restrict the scope of available candidate arcs, reduce the space of candidate solutions, and induce ants to avoid many unnecessary searches. Then, by combining the global score increase of a solution with the local anatomical constraint information between two corresponding ROIs, a new heuristic function with a better search ability is given to induct the process of ant stochastic searches. We then develop a new heuristic function with a better search ability to induct the process of ant stochastic searches. The experimental results on generated data and real fMRI-DTI datasets show that the new algorithm is more effective and efficient in identifying EC, and greatly enhances the convergence speed and learning quality compared to ACOEC and some other methods that only use single modality data.

## 2. Related Works

### 2.1. Ant Colony Optimization (ACO)

Ant colony optimization (ACO) is a meta-heuristic search algorithm inspired by the ant foraging theory. Ants use pheromones to communicate with each other in their feeding process. The more pheromones released on a route, the greater the probability is of ants selecting that route, which means that the more pheromones deposited on the shorter path over equal periods of time is, the greater the number of ants selecting the shorter path becomes. Thus, when one ant finds a very short path, other ants are more likely to follow this path. Such information feedback eventually leads all ants to select and follow the shortest path. In detail, each ant finds a solution starting from a start node and moving to feasible neighbor nodes step-by-step to construct a new solution. In the meantime, pheromones also evaporate over time during the process. For infrequently traveled paths, pheromone trails become weaker, and vice versa.

### 2.2. ACO for Learning Brain Effective Connectivity (ACOEC)

ACOEC (Liu et al., 2016) employs ACO to search for the best EC network, and takes each EC network as a directed acyclic graph (DAG) just like other methods based on BNs. It views each ant in ACO as an available solution (an EC network), employs a K2 scoring metric to evaluate each ant in a population, and guides ants to construct and search for the global maximum with the best K2 score in a feasible solution space.

In ACOEC, each ant *k* starts from an empty graph *G*(0) including all nodes (ROIs) and no arc, and proceeds by adding an arc at a time, and this process will be repeatedly performed until there is no way to make the score of the candidate solution higher by adding an arc. At time *t*, the probabilistic transition rule that an ant *k* selects a directed arc *a*_*ij*_ between two ROIs *X*_*i*_ and *X*_*j*_ from the current set of candidate arcs is defined as:

(1) $$\begin{array}{l} a_{i,j}=\{\begin{array}{rr} \; & arg\;max_{i,j\in DA_{k}(t)}\{[\tau_{ij}(t)]\cdot {\left[\eta_{ij}(t)\right]}^{\beta }\},\;if\;q\leq q_{0},\\ a_{I,J}, & otherwise, \end{array} \end{array}$$

where τ_*ij*_(*t*) is the pheromone concentration, η_*ij*_(*t*) represent the heuristic information of *a*_*ij*_, and β is the weighted coefficient which controls η_*ij*_(*t*) to influence the selection of arcs. *DA*_*k*_(*t*) (*i, j* ∈ *DA*_*k*_(*t*)) is the set of all candidate arcs whose heuristic information is larger than zero; *q*_0_ (0 ≤ *q*_0_ < 1) is an initial parameter that determines the relative importance of exploration vs. exploitation; *q* is a random number uniformly sampled in [0,1]; and *I* and *J* are a pair of ROIs randomly selected according to the probability in the following way:

(2) $$\begin{array}{l} p_{ij}^{k}(t)=\{\begin{array}{rr} \; & \frac{{\left[\tau_{ij}(t)\right]}^{\alpha }\cdot {\left[\eta_{ij}(t)\right]}^{\beta }}{\sum_{r,l\in DA_{k}(t)}{\left[\tau_{rl}(t)\right]}^{\alpha }\cdot {\left[\eta_{rl}(t)\right]}^{\beta },\;}\;if\;i,j\in DA_{k}(t),\\ 0, & otherwise, \end{array} \end{array}$$

where α denotes the relative importance of τ_*ij*_(*t*) left by ants. The heuristic function η_*ij*_ is defined as follows:

(3) $$\begin{array}{l} \eta_{ij}\left(t\right)=\omega \cdot \left(f\left(X_{i},Pa\left(X_{i}\right)\cup X_{j}\right)-f\left(X_{i},Pa\left(X_{i}\right)\right)\right), \end{array}$$

where *f*(*X*_*i*_, *Pa*(*X*_*i*_)) is the K2 score of the initial structure while *f*(*X*_*i*_, *Pa*(*X*_*i*_) ∪ *X*_*j*_) is the K2 score of new structure by adding an arc *X*_*j*_ → *X*_*i*_, ω = 1 + *Inf*(*X*_*i*_, *X*_*j*_) is a weighted factor associated with the arc connecting intensity, and *Inf*(*X*_*i*_, *X*_*j*_) represents the mutual information between *X*_*i*_ and *X*_*j*_.

For τ_*ij*_(*t*), ACOEC respectively carries out two pheromone updating processes. Moreover, once the iterations of ant colony searching end, the algorithm gets the optimal solution *G*^+^, i.e., EC network with the highest k2 score, and calculates the connection strength for every arc in *G*^+^.

## 3. The ACOEC-FD Algorithm

To enhance the performance of ACOEC, we employ two new strategies to learn the EC network from fMRI and DTI data.

### 3.1. Main Idea

**Figure 1:**
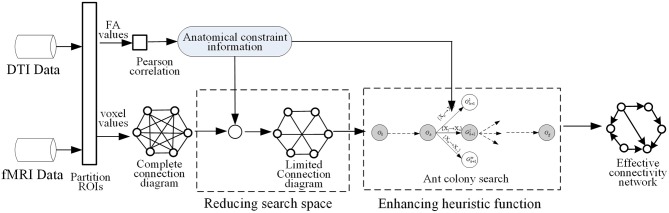
The main process of learning an effective connectivity network by ACOEC-FD.

As mentioned in some studies, the functional dynamics of a brain region is closely related to the pattern of its anatomical connections (Honey et al., 2007; van den Heuvel et al., 2009), and the anatomical fiber properties contribute to dynamic connectivity among homologous brain regions (Honey et al., 2009). Higher values of functional coherence in particular are linked to stronger AC (Xue et al., 2015). Motivated by such prior research, ACOEC-FD first uses DTI data to acquire anatomical constraint information, and then applies it to reduce the search space of the ant colony, which makes a candidate complete connection diagram become a limited connection diagram with smaller connections. Second, ACOEC-FD re-uses the obtained information to revise the heuristic function and to induct ants searching in the reduced space fast. The main process of ACOEC-FD is shown in Figure 1.

### 3.2. Acquiring Anatomical Constraint Information

First, we use FMRIB Software Library (FSL) to perform DTI data processing of multiple subjects, employ automated anatomical labeling (AAL) to partition the brain into *N* ROIs, and obtain the mean fractional anisotropy (FA) value of each ROI for every subject. We then calculate the Pearson correlation coefficient for each pair of ROIs *X*_*i*_ and *X*_*j*_ as follows:

(4) $$\begin{array}{l} r\left(X_{i},X_{j}\right)=\frac{1}{n-1}\overset{n}{\underset{l=1}{\sum }}\left(\frac{X_{i}^{l}-\bar{X_{i}}}{\delta_{X_{i}}}\right)\left(\frac{X_{j}^{l}-\bar{X_{j}}}{\delta_{X_{j}}}\right), \end{array}$$

where *n* is the number of DTI samples, $\bar{X_{i}}$ and δ_*X*_*i*__ are respectively the FA mean and standard deviation of *X*_*i*_. *r*(*X*_*i*_, *X*_*j*_) describes the intensity of linear correlation between two ROIs, and its range is [−1, 1] where *r*(*X*_*i*_, *X*_*j*_) > 0 show that *X*_*i*_ and *X*_*j*_ are positively correlated by means of FA values. The greater the value of *r*(*X*_*i*_, *X*_*j*_), the stronger the anatomical connection strength between *X*_*i*_ and *X*_*j*_. Based on all positively correlated relations, we build the adjacency matrix of the network of brain structures, and take it as anatomical constraint information to carry out two following processes in ACOEC-FD.

### 3.3. Reducing Search Space by Using Anatomical Constraint Information

The ACOEC algorithm is an iterative optimization algorithm, which stochastically searches for the optimal solution from all feasible solutions. In ACOEC, each ant selects a satisfied arc from a candidate connection diagram at each step in an iteration, thus the complexity of the candidate connection diagram determines the complexity of ACOEC to a large extent. Furthermore, if some useful strategies are developed to simplify the candidate connection diagram, then the search space of solutions will be greatly reduced. In light of the idea of that AC is necessary for EC, ACOEC-FD first uses anatomical constraint information obtained to reduce the search space before ants search. More specifically, we remove some redundant connections that do not satisfy the anatomical constraints based on DTI data from the initial connection diagram, and change the complete connection diagram to a limited connection diagram.

**Figure 2:**
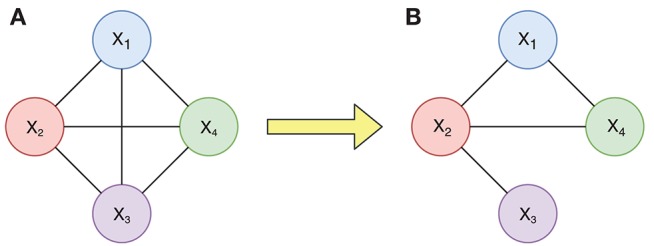
Changes of a connection diagram with 4 nodes. **(A)** Candidate connection diagram with four nodes. **(B)** Limited connection diagram with only four corresponding connections.

For example, we consider the changes of a candidate connection diagram with four nodes in Figure 2. Figure 2A is a complete connection diagram built on four ROIs, which has all six connections. If we use DTI data to acquire the anatomical constraint information *r*(*X*_1_, *X*_2_) > 0, *r*(*X*_2_, *X*_4_) > 0, *r*(*X*_2_, *X*_3_) > 0, and *r*(*X*_1_, *X*_4_) > 0, then we will obtain a limited connection diagram with only four corresponding connections, shown in Figure 2B.

**Figure 3:**
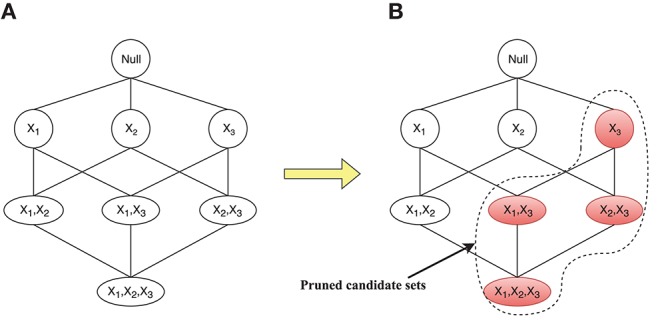
Corresponding changes of the search space for the candidate parent node set for *X*_4_. **(A)** Search space equal to the whole state space. **(B)** Search space is reduced to four candidate parent node sets.

The different initial connection diagrams will directly determine the size of search spaces of some nodes at different iterations. Corresponding to the connection diagrams in Figure 2, the changes in search spaces for the parent set of *X*_4_ are given in Figure 3. Figure 3A shows the search space equal to the whole state space when there are connections between all *X*_4_ and other nodes, and Figure 3B depicts that the search space is greatly reduced to four candidate parent node sets {}, {*X*_1_}, {*X*_2_}, and {*X*_1_, *X*_2_}. Since the connection between *X*_1_ and *X*_4_ fails to satisfy the anatomical constraint, those candidate parent node sets for *X*_4_, shaded in Figure 3B, could be pruned.

Using this strategy, the connections that failed to satisfy the anatomical constraints, will be prevented from being constructed by ants, thus the search spaces for many nodes would be greatly reduced, which would reduce the computational time required for ACO to learn EC.

### 3.4. Revising Heuristic Function by Reusing Anatomical Constraint Information

In ACOEC, the heuristic function is defined as the product of the connecting intensity of an arc and the score increase introduced by adding an arc, which integrates the global information (score increase) with the local information (arc connecting intensity) and guides an ant to select arcs. However, the definition has a drawback, in that it only provides the heuristic information of arcs from the view of FC, and overlooks other relevant and available information. That makes the heuristic information unilateral and might influence the searching ability of the heuristic function. Considering that some study results show that stronger levels of EC (derived from fMRI data) are associated with stronger levels of AC (derived from DTI data), the new heuristic function of a directed arc is defined as:

(5) $$\begin{array}{l} \eta_{ij}\left(t\right)=\omega \cdot \omega^{\prime }\cdot \left(f\left(X_{i},Pa\left(X_{i}\right)\cup X_{j}\right)-f\left(X_{i},Pa\left(X_{i}\right)\right)\right), \end{array}$$

where a new weighted factor $\omega^{\prime }=1+r\left(X_{i},X_{j}\right)$ is concerned with the anatomical connecting intensity of the corresponding arc, and *r*(*X*_*i*_, *X*_*j*_) > 0 represents the arc *a*_*ij*_ to be positively correlated. *r*(*X*_*i*_, *X*_*j*_) > 0 reflects whether the two nodes are correlated and how much that correlation is; thus, it can also be used as heuristic information to induce ant selecting arcs. A new heuristic function employs the local correlation information of arcs from DTI to participate in selecting an arc. Obviously, when two connecting intensities of an arc from both fMRI and DTI data are strong, and the score increase by adding the arc is large, the heuristic function value is great, and vice versa. That is, such arcs, which not only have significant function connecting intensities on fMRI but also have strong anatomical connecting evidence on DTI, are more likely to be explored. Because the revision of the heuristic function can cause ACO to avoid some unnecessary explorations for some structures that lack anatomical connectivity evidence, this would also reduce the computational time required for ACO to learn EC.

### 3.5. Algorithm Description

In summary, ACOEC-FD employs two strategies, combining fMRI with DTI, to improve ACOEC. First, the Pearson correlation coefficient computing is introduced so that anatomical constraint information from DTI is exploited to restrict the search space, thus avoiding some unnecessary searches. Second, the anatomical constraint information from DTI is re-used in the new heuristic function, enhancing the purpose of construction solutions during ant random searching (i.e., heuristic ability). In other words, ACOEC-FD not only makes use of anatomical constraint information to reduce the search space, but also takes it as other heuristic information to induce random searching. In contrast to ACOEC, there are two main differences: (1) using Pearson correlation coefficient computing to obtain a limited connection graph so that the search space is greatly reduced; (2) combining anatomical constraint information base on DTI with the functional connectivity information (mutual information and score increase) based on fMRI to propose a more powerful heuristic function. In fact, effective connectivity can exist without underlying anatomical connections. However, some important connections may be ignored as the search space is restricted to the regions that possess anatomical connections. To overcome this issue, we perform local optimization when all ants finish their searching. The local optimization operation can get the effective connectivity under the situation when there is no anatomical connection between two brain regions. In detail, if an arc is important and has a higher K2 score, the arc will be added even if there is no anatomical connection. Finally, the termination condition of the ACOEC-FD is that once the algorithm obtains the same optimal solution for 10 successive generations, the search phase will end.

Algorithm 1 provides the main processes of ACOEC-FD.

**Algorithm 1:**
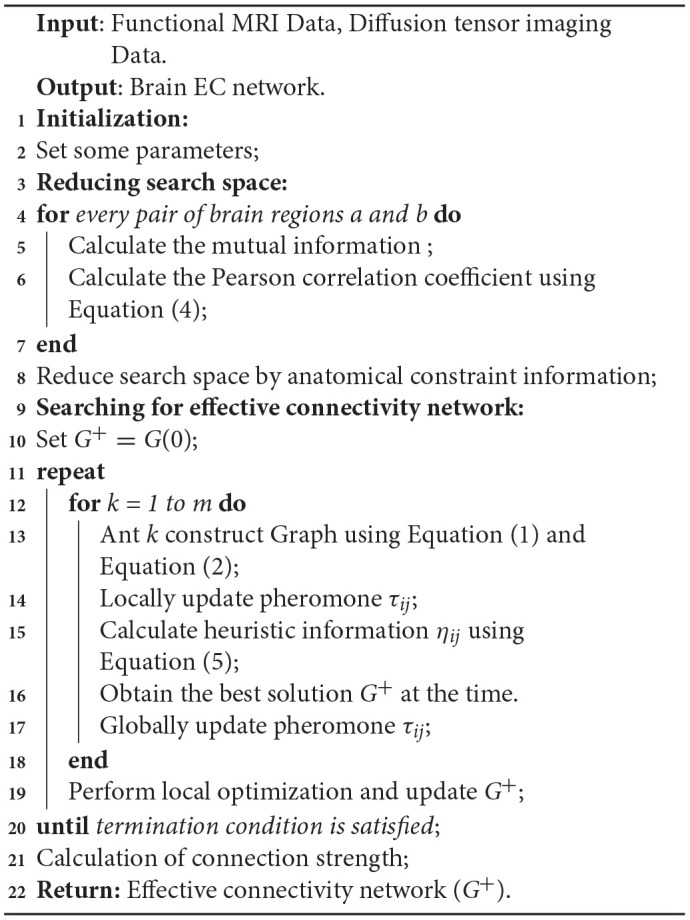
ACOEC-FD

### 3.6. Algorithm Analysis

The main cost of the ACO algorithm is the computation of statistic factors. Let *L* be the number of iterations, *m* be the number of ant colony, and *N* be the number of ROIs, the complexity of ACOEC can be simply summarized as *O*(*N*^2^) + *O*(*L* · *m* · *N*^2^) + *O*(*N*^2^) + *O*(1) ≈ *O*((*L* · *m* + 2) · *N*^2^) according to its processes. In contrast to ACOEC, ACOEC-FD performs two changes in the first two processes, thus the complexity level is the same as ACOEC though the complexity of the initialization process slightly increases from *O*(*N*^2^) to *O*(3 · *N*^2^). Notably, ACOEC-FD first employs anatomical constraint information to reduce the search space, which will greatly decrease candidate connections (*N*^2^), and then merges anatomical constraint information into the heuristic function to induct the process of stochastic searches, which may effectively decrease the number of iterations (*L*).

## 4. Experimental Results

To assess the performance of ACOEC-FD, we first use a common evaluation method, which is to test the algorithm and its new strategies on a set of simulated fMRI and DTI data, generated from known ground-truth networks. Then, to illustrate the application potential of ACOEC-FD, we apply it to real AD datasets to discriminate EC differences between four subject groups. The experimental platform is a PC with Intel (R) Core (TM) i7-4770, 3.40GHz CPU, 16 GB RAM, and Windows 7. Codes are available at https://github.com/teddyduo/teddyduo.

### 4.1. Datasets

#### 4.1.1. Simulation Datasets

The generation model of simulations is referenced in the methods of Smith et al. (2011), which used the dynamic causal modeling (DCM) to generate the neural timeseries. The neural network model is shown as:

(6) $$\begin{array}{l} ż=\sigma Az+Cu, \end{array}$$

where *z* is the neural timeseries, ż is its rate of change, *u* is the external inputs (gaussian noises), *C* is the external input matrix where only diagonal elements are 1 and others are 0, and the matric *A* determines the network connections between nodes which indicate the ground-truth. The sizes of the matrix *C* and *A* are *N* × *N*, and σ controls the neural lag between ROIs.

**Figure 4:**
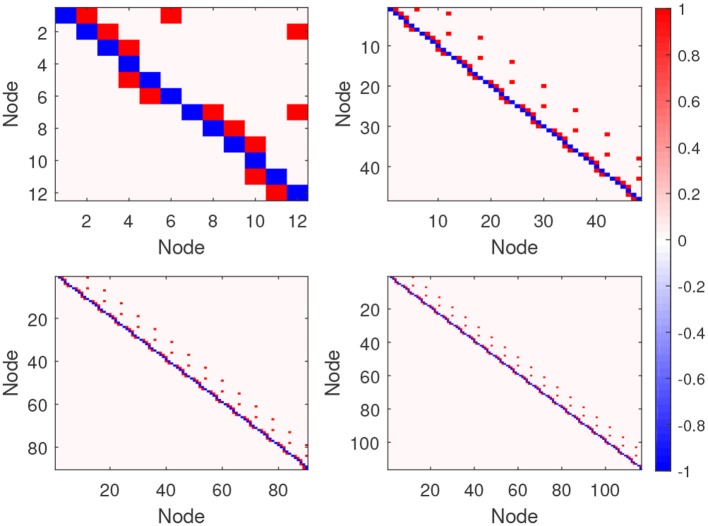
The connection matrix of the four ground-truths on the four simulation datasets: The upper left is Sim1, upper right is Sim2, lower left is Sim3, and lower right is Sim4. For each network graph the corresponding connection matrix is shown, where an element in the upper diagonal of the matrix (red gird) implies a directed connection from a lower-numbered node to a higher-numbered one, and an element in the lower diagonal of the matrix (red gird) implies a directed connection from a higher-numbered node to a lower-numbered one.

The BOLD fMRI timeseries are obtained from the neural timeseries after convolution with a hemodynamic response function (HRF). We set the session duration to 10 min, and the BOLD fMRI timeseries are sampled with a TR of 3 s, so the number of timepoints is 200. Based on different ROIs, we generate four simulated fMRI datasets, namely Sim1, Sim2, Sim3, and Sim4, whose number of ROIs are 12, 48, 96, and 116, respectively. Each simulation comprises 50 separate subjects, and all of these subjects use the same simulation parameters. Additionally, we also generated a set of anatomical connections corresponding to the effective connections in the four simulations. Synthetic anatomical connections corresponding to no dynamic influence are randomly given zero probability, or one, because the presence of anatomical connections does not determine effective connectivity, which is dynamic in nature (Dang et al., 2017). The ground-truth of the four simulations are shown in Figure 4, where each connection matrix corresponding to an effective connectivity network is shown. In detail, a value of 1 means there is a directed connection between these two nodes.

#### 4.1.2. Real Alzheimer's Disease Datasets

Real data used in this article was obtained from the ADNI database (adni.loni.usc.edu). For details, please see http://www.adni-info.org.

In this study, we used 35 subjects from the ADNI database presenting concurrently volumetric *T*1-weighted (T1-w), DTI and fMRI data. These subjects belong to four groups according to ADNI baseline diagnosis: healthy controls (HC), and early MCI (EMCI), late MCI (LMCI), and AD patients. The subject selection criteria are as follows: The session duration and time points of each subject's fMRI data are the same, furthermore, for each subject, the MRI (contains T1-w and fMRI data) and DTI data were obtained at the same time. The characteristics of the subjects are shown in Table 1.

**Table 1:** The characteristics of the HC, EMCI, LMCI, and AD.

	**HC**	**EMCI**	**LMCI**	**AD**
*Num*.^a^	10	10	10	5
Gender^b^	8F/2M	3F/7M	4F/6M	3F/2M
Mean Age^c^	75.3 ± 7.7	78.7 ± 5.0	80.0 ± 9.0	74.1 ± 12.0
Age Range^d^	[65–87]	[70–86]	[68–96]	[56–89]

a *Number of subjects*.

b *F, Female; M, Male*.

c *The mean age ± standard deviation*.

d *The age range (years old)*.

Neuroimaging MRI data including high-resolution fMRI data and DTI data was acquired using a 3T MRI scanner (Siemens) and an 8-channel receive only head coil. fMRI sequence parameters include: Slices = 48; volumes = 140; TR/TE = 3,000/30 ms; FA = 90°; Matrix = 64×64. DTI sequence (spin echo with echo planar readout): axial plane; Slices = 80; gradient directions = 54.0; TR/TE = 7,200/56 ms; FA = 90°; Slice thickness=2.0 mm, Reconstruction matrix = 256×256; b = 1,000 *s*/*mm*^2^. For more data acquisition information, please see http://www.adni-info.org.

fMRI data preprocessing was performed using the Data Processing Assistant for Resting-State fMRI (DPARSF, http://www.restfmri.net), which is based on Statistical Parametric Mapping (SPM8, http://www.fil.ion.ucl.ac.uk/spm) and Resting-State fMRI Data Analysis Toolkit (REST). The steps used for preprocessing are as follows: (1) Arrange the DICOM files and discard the first 10 time points of each session; (2) Set parameters, DPARSF will then give all the preprocessed (slice timing, realign, normalize, smooth) data; (3) Define ROIs and get fMRI time series data. In this paper, we employed two methods of dividing ROIs: (1) Use a brain template to parcellate the whole cortex into multiple brain regions, and treat each brain as a ROI, e.g., we utilize the AAL template to achieve 116 ROIs; (2) Decompose several independent brain networks from fMRI data, and define these specific networks as ROIs, e.g., we define some ROIs from the Default Mode Network (DMN) and the Executive Control Network (ECN) based on the literature.

DTI data were preprocessed using the FSL toolbox (https://fsl.fmrib.ox.ac.uk/fsl/fslwiki/FSL). The steps are as follows: (1) Transform raw DICOM images into NIFTI format using dcm2nii; (2) Create a mask using brain extraction which segments the brain from the skull and other extracranial structures; (3) Correct the head motion and eddy current distortion of images; (4) Utilize the brain template (same with the fMRI data) to define ROIs, and get the FA value for every ROI by computing the averaging FA of all fibers inside each region.

After fMRI data preprocessing, the preprocessed fMRI data also requires discrete processing. The reason is that ACOEC-FD employs the K2 metric, which requires data discretization. According to the number of time points, the discretized instance data are obtained for the whole brain, where each instance includes the discretized values of all brain regions (nodes) at the corresponding time point. For each node's timeseries of a subject, the range of voxel values is divided into several equal parts, thus each part contains the same number of voxel values. Then the voxel value of each node is quantized at every instance into a discrete value. For instance, a node's timeseries is quantized into five parts, including very low (set value = 0), low (set value = 1), medium (set value = 2), high (set value = 3), and very high (set value = 4), with each of the five parts containing 20% of the data points.

### 4.2. Evaluation Metrics

We compared the learned results to the ground-truths on four common graph metrics (Ji et al., 2016; Liu et al., 2016; Zheng et al., 2018): (1) Precision (PRE), (2) Recall (REC), (3) F1-measure (F1), (4) Structural Hamming distance (SHD). In detail, SHD is the total number of edge additions, deletions, and reversals needed to convert the learned effective connectivity network into the ground-truth network. Let *L*_*net*_ denote the learned network and *G*_*net*_ express the ground-truth network. The four evaluation metrics are then given by:

(7) $$\begin{array}{l} PRE=\frac{SD}{TD_{L_{net}}}, \end{array}$$

(8) $$\begin{array}{l} REC=\frac{SD}{TD_{G_{net}}}, \end{array}$$

(9) $$\begin{array}{l} F1=\frac{2*PRE*REC}{PRE+REC}, \end{array}$$

(10) $$\begin{array}{l} SHD=RD+FD+MD, \end{array}$$

where *SD* represents the number of same directed arcs (arcs are both in *L*_*net*_ and *G*_*net*_), *RD* represents the number of reversed directed arcs (arcs have different directions between *G*_*net*_ and *L*_*net*_), *FD* represents the number of false arcs (arcs are not in *G*_*net*_ but in *L*_*net*_), *MD* represents the number of missing arcs (arcs are not in *L*_*net*_ but in *G*_*net*_), *TD*_*L*_*net*__, and *TD*_*G*_*net*__, respectively denote the total number of directed arcs in *L*_*net*_ and *G*_*net*_.

### 4.3. Contributions of Two New Strategies

**Figure 5:**
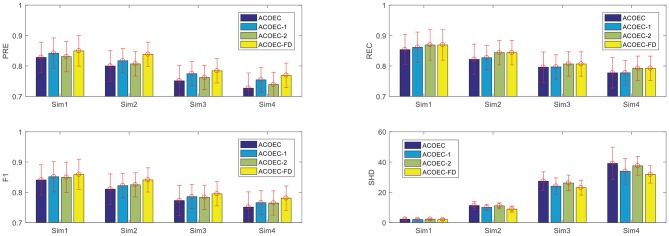
Contributions of two new strategies for ACOEC. Horizontal axis corresponds to the four simulation data sets, vertical axis corresponds to the four measurements on each simulation. Error bars represent standard deviation for the given metric over 10 runs.

First, to quantitatively examine the effectiveness and efficiency of two new strategies, we employed four algorithms to learn EC structures from simulated data sets with different node scales. The four algorithms are respectively the original ACOEC, an improved ACOEC-1 (only add reducing search space with anatomical constraint information), another improved ACOEC-2 (only add revising the heuristic function with anatomical constraint information), and ACOEC-FD with two new strategies. The experimental results on four datasets are shown in Figure 5, where the performance of the algorithms is evaluated using four measures: Precision (PRE), Recall (REC), F1-measure (F1), and Structural Hamming distance (SHD).

In the following section, we analyze how the two new strategies contribute to ACOEC algorithm on different simulations. From Figure 5, we can see that the two strategies can slightly increase the PRE, REC, and F1 values of the ACOEC algorithm, and the improving rate is about 1 to 6%. More specifically, ACOEC-1 employs anatomical constraint information to effectively reduce the search space, which increases the possibility that ants obtain a better solution. ACOEC-2 uses the new heuristic function with anatomical constraint information to enhance the ant random search ability to some extent. The lower SHD values for three improved algorithms show that both of the two strategies can make the ACOEC algorithm have fewer incorrect arcs. Specifically, effectively reducing the search space may decrease the likelihood of an unexpected arc addition, while the new heuristic function with anatomical constraint information plays a better role in determining the directions of arcs. This result indicates that two strategies can improve the SHD performance of the ACOEC algorithm from two different point of view.

Furthermore, we compared the time performance of the four algorithms, focusing on the running time to learn a brain EC network. The reported results are summarized in Table 2, where the μ±σ indicates the mean μ and the standard deviation σ over 10 executions independently carried out by the corresponding algorithm. It is important to note that the two strategies can significantly improve the convergence performance. By reducing the search space, ACOEC-1 greatly decreases the running time compared to ACOEC. In particular, when the node number is larger, e.g., 48, 90, and 116, the strategy becomes more efficient. The reduced time is 120, 771, and 2,372 s, and the improving ratios are about 54, 59, and 94%, respectively. Moreover, the new heuristic function also improves the convergence performance while maintaining a better solution quality. The reduced time is 0.26, 14, 320 and 1,411 s for four cases, respectively. The main reason is that anatomical constraint information is integrated into the heuristic function to make the arc selection more reasonable, which reduces the number of iterations.

**Table 2:** Contributions of two new strategies on time performance.

**Data (nodes)**	**Algorithms**	**Times (s)**
	ACOEC	3.92 ± 0.25
Sim1 (12)	ACOEC-1	2.97 ± 0.18
	ACOEC-2	3.66 ± 0.21
	ACOEC-FD	3.38 ± 0.19
	ACOEC	270.36 ± 9.20
Sim2 (48)	ACOEC-1	149.49 ± 4.18
	ACOEC-2	256.71 ± 8.20
	ACOEC-FD	123.04 ± 3.13
	ACOEC	1411.62 ± 144.96
Sim3 (90)	ACOEC-1	639.76 ± 16.87
	ACOEC-2	1091.15 ± 90.42
	ACOEC-FD	577.34 ± 87.63
	ACOEC	3342.85 ± 115.52
Sim4 (116)	ACOEC-1	968.67 ± 70.41
	ACOEC-2	1931.10 ± 87.45
	ACOEC-FD	796.57 ± 27.19

The results in Figure 5 and Table 2 show that based on anatomical constraint information, ACOEC-FD integrates two strategies of reducing the search space and revising heuristic function into ACOEC, which can not only greatly accelerate the process of learning EC networks but can also effectively improve the learning results in all cases.

### 4.4. Comparing ACOEC-FD With Other Algorithms

To illustrate ACOEC-FD's ability to accurately infer EC, we compare it with another 10 algorithms only using fMRI data. These algorithms are the LiNGAM, ParceLiNGAM (Tashiro et al., 2014), pairwise LiNGAM (PWLiNGAM) (Hyvärinen and Smith, 2013), GC, GS, Patel, GES, P-corr, AIAEC, and the ACOEC algorithm, respectively. They are classic methods used to identify brain EC networks, some of them perform well on Smith's simulated dataset, and some of them are state-of-the-art methods. The parameters of the algorithms under comparison are based on existing literature (Ji et al., 2016; Liu et al., 2016). The default parameter configurations of the corresponding methods are as follows. LiNGAM uses the parameters where *Prune Factor* = 1.0. ParceLiNGAM runs with *Alpha* = 0.05. PWLiNGAM is performed with *method* = 1. GC is set as *max*_*lag* ∈ [1, 30], *Alpha* = 0.05. GS is performed with *tau* = 2, *m* = 10, *nn* = 10, *and theiler* = 50. Patel runs with *bin* = 0.75. The parameters of GES is set as *PenaltyDiscount* = 1.0, and *NumPatternstoSave* = 1. P-corr runs with *BOLDMaxlength* = 15, *TR* = 3. The AIAEC is set as *P*_*s*_ = 0.5, *P*_*c*_ = 0.6, *P*_*m*_ = 0.4, *T* = 150, *N* = 80, and *M* = 70. ACOEC and ACOEC-FD use the parameters where α = 1, β = 2, ρ = 0.2, *q*_0_ = 0.8, *a* = 10, *NC* = 100, *l*_*step*_ = 10. AIAEC, ACOEC, and ACOEC-FD are random optimization methods, whose results are not always same during each run, so we show the mean μ and the standard deviation σ results over 10 random runs. If the standard deviation is zero, we only show the mean value.

In our experiments, we compared 11 different algorithms, to illustrate which one is the best one. We ran them on four simulation datasets, and the detailed results are shown in Table 3. From Sim1 to Sim4 in Table 3, the number of nodes increased from 12, to 48, 90, and 116. Following the chain of Sim1-Sim2-Sim3-Sim4, we can see that most of the algorithms including ACOEC-FD decrease very little on four evaluated metrics. However, three swarm intelligence-based algorithms (AIAEC, ACOEC, and ACOEC-FD) obtained prominent results compared to those of another eight algorithms. Compared to AIAEC and ACOEC, ACOEC-FD has a better and more reliable performance when the number of nodes increases. In Sim4, the ACOEC-FD's PRE, REC, and F1 value retain better values (0.77, 0.79, and 0.78) which are higher than those of the other 10 algorithms, while SHD mean value is 31.8, which is the smallest value among all 11 algorithms. Therefore, most of the algorithms do not perform well when the number of nodes is large, however, ACOEC-FD using fMRI and DTI data can still achieve good performance and have fewer mistakes.

**Table 3:** Comparisons of 11 algorithms on the four simulations.

**Sim**	**Metrics**	**Algorithms**										
		**LiNGAM**	**ParceLiNGAM**	**PWLiNGAM**	**GC**	**GS**	**Patel**	**GES**	**P-corr**	**AIAEC**	**ACOEC**	**ACOEC-FD**
Sim1	PRE	0.57	0.77	0.85	0.61	0.62	0.85	0.69	0.85	0.84 ± 0.06	0.83 ± 0.07	0.85 ± 0.06
	REC	0.62	0.77	0.85	0.85	0.62	0.85	0.69	0.85	0.85 ± 0.06	0.85 ± 0.06	0.87 ± 0.06
	F1	0.59	0.77	0.85	0.71	0.62	0.85	0.69	0.85	0.84 ± 0.06	0.84 ± 0.06	0.86 ± 0.06
	SHD	6	3	2	7	5	2	4	2	2.2 ± 0.72	2.3 ± 0.74	1.8 ± 0.67
Sim2	PRE	0.54	0.69	0.76	0.52	0.58	0.73	0.59	0.54	0.80 ± 0.08	0.80 ± 0.08	0.83 ± 0.07
	REC	0.64	0.73	0.80	0.76	0.65	0.82	0.64	0.56	0.81 ± 0.08	0.82 ± 0.08	0.84 ± 0.07
	F1	0.58	0.71	0.78	0.62	0.62	0.77	0.61	0.55	0.81 ± 0.08	0.81 ± 0.08	0.84 ± 0.07
	SHD	30	18	14	39	26	17	24	26	11.6 ± 4.62	11.3 ± 4.55	8.9 ± 4.02
Sim3	PRE	0.51	0.66	0.71	0.47	0.53	0.69	0.59	0.56	0.76 ± 0.08	0.75 ± 0.08	0.78 ± 0.07
	REC	0.61	0.71	0.76	0.75	0.63	0.78	0.63	0.58	0.80 ± 0.08	0.80 ± 0.07	0.81 ± 0.07
	F1	0.55	0.69	0.73	0.58	0.58	0.73	0.61	0.57	0.78 ± 0.08	0.77 ± 0.07	0.80 ± 0.07
	SHD	61	38	33	88	57	37	45	48	26.4 ± 8.05	26.8 ± 8.08	23.1 ± 6.88
Sim4	PRE	0.50	0.62	0.69	0.48	0.52	0.67	0.56	0.54	0.74 ± 0.08	0.73 ± 0.09	0.77 ± 0.08
	REC	0.59	0.69	0.76	0.67	0.60	0.75	0.60	0.56	0.78 ± 0.08	0.78 ± 0.08	0.79 ± 0.08
	F1	0.54	0.65	0.72	0.56	0.56	0.71	0.58	0.55	0.75 ± 0.08	0.75 ± 0.08	0.78 ± 0.08
	SHD	80	56	46	96	75	50	64	64	39.1 ± 10.16	39.3 ± 10.42	31.8 ± 7.29

**Figure 6:**
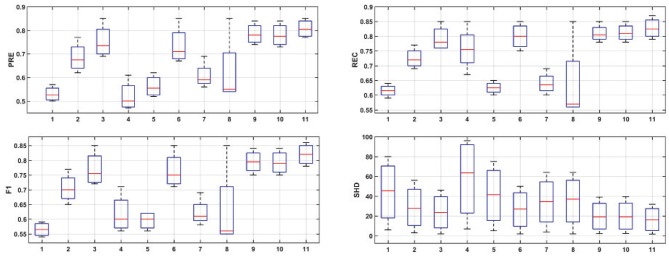
Results for simulation experiments of 11 algorithms. The horizontal axis corresponds to the eleven algorithms, which are LiNGAM (1), ParceLiNGAM (2), PWLiNGAM (3), GC (4), GS (5), Patel (6), GES (7), P-corr (8), AIAEC (9), ACOEC (10), and ACOEC-FD (11), respectively. The vertical axis corresponds to the four measurements.

Figure 6 provides the box plots of PRE, REC, F1, and SHD comparisons for the 11 algorithms on four simulations. It is clear that ACOEC-FD achieved the best performance whether on PRE, REC, F1, or SHD compared to the other algorithms. As shown in Figure 6, we also found that GC performed worse on PRE and SHD but performed better on REC. This is mainly because GC always has a lot of extra adding arcs, which influences the PRE and SHD metrics. P-corr performed badly on PRE, REC and F1 metrics, but its SHD value was not too high. This is because P-corr has extremely few adding and missing arcs though it has a lot of reverse arcs from the comparison between the *L*_*n*_*et* and *G*_*n*_*et*. This result shows that different metrics can evaluate the performance of algorithms from different angles. However, in all evaluated metrics, ACOEC-FD performed the best, and the mean of each metric is significantly better than the other algorithms.

**Figure 7:**
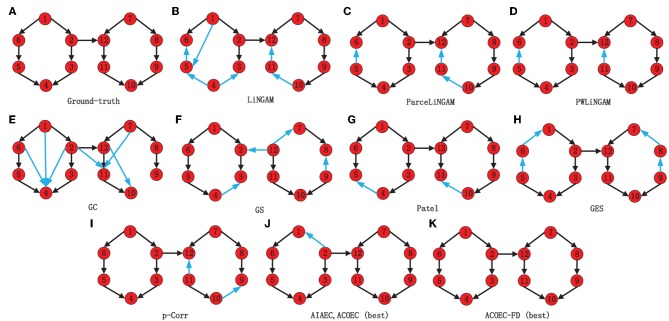
Comparison of *G*_*net*_ and *L*_*net*_ by 11 different algorithms on Sim1. The eleven algorithms, which are the Ground-truth **(A)**, LiNGAM **(B)**, ParceLiNGAM **(C)**, PWLiNGAM **(D)**, GC **(E)**, GS **(F)**, Patel **(G)**, GES **(H)**, P-corr **(I)**, AIAEC and ACOEC **(J)**, and ACOEC-FD **(K)**, respectively. In each graph, black lines mean that the connections and directions in this graph are consistent with the *G*_*net*_, while the blue lines are not.

In order to present the results more clearly and intuitively, we provide the network structure diagram learned by various algorithms on Sim1 as an instance. Figure 7 shows a comparison of *G*_*net*_ and *L*_*net*_ by 11 algorithms, where (a) is *G*_*net*_, (b)-(k) are *L*_*net*_ by 11 algorithms, and the blue arcs in *L*_*net*_ are the error arcs identified. Obviously, the EC network identified by ACOEC-FD is completely consistent with *G*_*net*_ while there is at least one error arc in other *L*_*net*_ learned by other algorithms. This instance also verifies that ACOEC-FD can more accurately infer EC using multimodal Data.

Finally, we use the Friedman test and the *post-hoc* test to attest the efficiency of the corresponding algorithms. If the *p*-value obtained from the test is less than 0.05, we consider that a significant difference exists in the corresponding experimental results. The result of the Friedman test (*p*-value, chi-squared and degrees of freedom) is shown in Table 4, which indicates that there is a significant difference between the 11 compared algorithms on the four evaluate metrics. Therefore, we employ *post-hoc* test to find out which pairs of algorithms are significantly different from each other. The *post-hoc* test results on the PRE, REC, F1, and SHD are shown in Tables 5–**8**, respectively. Bold type signifies statistically significant results (*p*-value < 0.05).

**Table 4:** The Friedman test results of 11 algorithms on four metrics.

	***p*-value**	**Chi-squared**	**Degrees of freedom (df)**
PRE	1.2744 × 10^−4^	34.95	10
REC	1.2370 × 10^−4^	35.02	10
F1	2.0531 × 10^−4^	33.73	10
SHD	7.2612 × 10^−5^	36.37	10

**Table 5:** The *post-hoc* test results of *p*-values on PRE value.

	**ParceLiNGAM**	**PWLiNGAM**	**GC**	**GS**	**Patel**	**GES**	**P-corr**	**AIAEC**	**ACOEC**	**ACOEC-FD**
LiNGAM	0.8537	0.1613	1.0000	1.0000	0.3221	0.9786	0.9613	0.0795	0.1410	**0.0078**
ParceLiNGAM		0.9892	0.6955	0.9926	0.9993	1.0000	1.0000	0.9496	0.9845	0.5816
PWLiNGAM			0.0795	0.5036	1.0000	0.9003	0.9358	1.0000	1.0000	0.9969
GC				0.9988	0.1819	0.9193	0.8781	**0.0353**	0.0685	**0.0027**
GS					0.7315	1.0000	1.0000	0.3221	0.4657	0.0578
Patel						0.9786	0.9892	1.0000	1.0000	0.9709
GES							1.0000	0.7644	0.8781	0.2911
P-corr								0.8265	0.9192	0.3557
AIAEC									1.0000	0.9998
ACOEC										0.9980

From Table 5, we can find that AIAEC and ACOEC-FD are significantly better (*p*-value<0.05) than GC, and the *p*-values are 0.0353 and 0.0027, respectively. Besides, only the ACOEC-FD algorithm is significantly better than LiNGAM (*p*-values = 0.0078 < 0.05). This result indicates that the ACOEC-FD algorithm has more outstanding results on the PRE metric compared with other algorithms.

Table 6 shows that only the ACOEC-FD algorithm is significantly better than LiNGAM, GS, GES, and P-corr, and the *p*-values are 0.0038, 0.0242, 0.0242, and 0.0117, respectively. The results indicate that the ACOEC-FD algorithm perform well on the REC metric.

**Table 6:** The *post-hoc* test results of *p*-values on REC value.

	**ParceLiNGAM**	**PWLiNGAM**	**GC**	**GS**	**Patel**	**GES**	**P-corr**	**AIAEC**	**ACOEC**	**ACOEC-FD**
LiNGAM	0.9673	0.4003	0.7789	1.0000	0.2366	1.0000	1.0000	0.1244	0.0804	**0.0038**
ParceLiNGAM		0.9944	1.0000	0.9992	0.9673	0.9992	0.9944	0.8894	0.8106	0.2366
PWLiNGAM			1.0000	0.7455	1.0000	0.7455	0.5956	1.0000	0.9998	0.8894
GC				0.9673	0.9992	0.9673	0.9102	0.9915	0.9758	0.5551
GS					0.5551	1.0000	1.0000	0.3644	0.2661	**0.0242**
Patel						0.5551	0.4003	1.0000	1.0000	0.9673
GES							1.0000	0.3644	0.2661	**0.0242**
P-corr								0.2366	0.1642	**0.0117**
AIAEC									1.0000	0.9943
ACOEC										0.9986

From Table 7, we can see that the ACOEC-FD algorithm is significantly better than LiNGAM and GS. However, there is no significant difference between other algorithms. These results indicate that the ACOEC-FD algorithm has a better performance on the F1 metric.

**Table 7:** The *post-hoc* test results of *p*-values on F1 value.

	**ParceLiNGAM**	**PWLiNGAM**	**GC**	**GS**	**Patel**	**GES**	**P-corr**	**AIAEC**	**ACOEC**	**ACOEC-FD**
LiNGAM	0.6969	0.1076	0.9892	0.9981	0.1845	0.9847	0.9969	0.0422	0.0587	**0.0014**
ParceLiNGAM		0.9951	0.9993	0.9951	0.9993	0.9996	0.9969	0.9617	0.9788	0.4661
PWLiNGAM			0.7663	0.6223	1.0000	0.7979	0.6598	1.0000	1.0000	0.9788
GC				1.0000	0.8790	1.0000	1.0000	0.5446	0.6223	0.0801
GS					0.7663	1.0000	1.0000	0.3926	0.4661	**0.0422**
Patel						0.9006	0.7979	1.0000	1.0000	0.9360
GES							1.0000	0.5830	0.6598	0.0935
P-corr								0.4285	0.5050	0.0505
AIAEC									1.0000	0.9981
ACOEC										0.9951

The results in Table 8 clearly show that the AIAEC and ACOEC-FD algorithms are significantly better than GC, with *P*-values of 0.0256 and 0.0010, respectively. Additionally, only the ACOEC-FD algorithm is significantly better than LiNGAM and GS. From these experimental results, we can draw the conclusion that ACOEC-FD is superior to the other 10 algorithms on the four evaluate metrics and has a significant difference in some performances when compared to some algorithms.

**Table 8:** The *post-hoc* test results of *p*-values on SHD value.

	**ParceLiNGAM**	**PWLiNGAM**	**GC**	**GS**	**Patel**	**GES**	**P-corr**	**AIAEC**	**ACOEC**	**ACOEC-FD**
LiNGAM	0.8811	0.2109	1.000	1.000	0.3965	0.9895	0.9510	0.0948	0.1654	**0.0056**
ParceLiNGAM		0.9928	0.6265	0.9895	0.9997	1.000	1.000	0.9510	0.9850	0.4719
PWLiNGAM			0.0795	0.5036	1.0000	0.9003	0.9358	1.0000	1.0000	0.9969
GC				0.9981	0.1655	0.9023	0.7695	**0.0256**	0.0519	**0.0010**
GS					0.7359	1.0000	0.9981	0.2958	0.4335	**0.0312**
Patel						0.9792	0.9970	1.0000	1.0000	0.9211
GES							1.0000	0.7359	0.8568	0.1879
P-corr								0.8811	0.9510	0.3290
AIAEC									1.0000	0.9989
ACOEC										0.9928

### 4.5. Application of ACOEC-FD on Alzheimer's Disease

**Figure 8:**
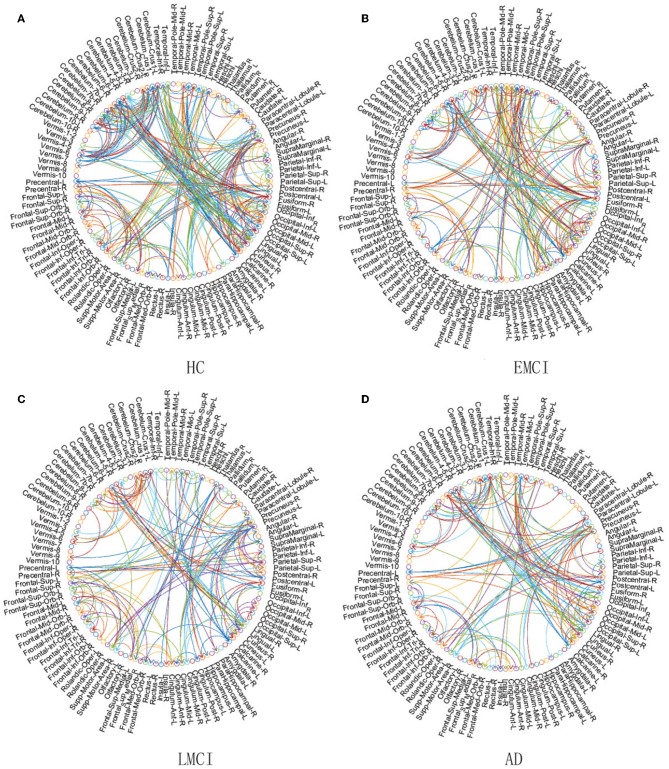
Comparison of four EC networks for HC **(A)**, EMCI **(B)**, LMCI **(C)**, and AD **(D)** groups on real fMRI data.

In this section, we present an application example that we use ACOEC-FD on, in four real Alzheimer's Disease datasets to discriminate EMCI, LMCI, and AD from HC. The EC networks learned for four different groups are graphically rendered in a circular diagram format in Figure 8. For each circular diagram, the outermost rings denote the brain regions and the lines in the center express the EC. The labels are achieved by the AAL template, which consists of 116 ROIs. Each brain regions is represented by a circle with differentiated colors (some may be the same), and the arrow colors are consistent with their parent nodes.

Following the chain of HC-EMCI-LMCI-AD in Figure 8, the EC number in each group is 356, 251, 205, and 191, respectively. That is, as the disease worsens, the EC number will be less and less. In detail, the number of reduced directions and the number of reversed directions between the HC and AD group are 165 and 58, respectively. In particular, there are significant decreases of EC in the Precuneus, Occipital, Precentral, Insula, and Hippocampus brain regions etc. These findings once again verify previous studies implicating that there are changes in these regions related to MCI and AD, which can help explain and predict the progression and evolution of AD disease (He et al., 2008; Ribeiro et al., 2015). From Figure 8 we find that the brain EC in the hippocampus has a significant reduction from the ECMI group to the LMCI group and to the AD group, and there are very few ECs in the AD group. The reason may be that the center of memory function is mainly located in the temporal lobe, especially in the hippocampus. Therefore, the reduction of the EC in the hippocampus directly leads to brain memory function degradation, causing people to have mild cognitive impairment. As the disease continues to worsen, the brain EC of the hippocampal region becomes less and less. In fact, structural decline usually occurs earlier than functional decline, so ACOEC-FD utilizes the probabilities of structural connections between regions as supplemental criteria for establishing these effective connectivity networks, whose constructed networks are more in line with the physiological structure of the human brain. Moreover, we find that some regions of the cerebellum in AD patients also show decreased EC compared to those of HC subjects, which is different from many results under single modal data (Wang et al., 2007; Qi et al., 2010). This phenomenon may be related to compensatory processes for MCI and AD patients, however, the implicative mechanism behind the phenomenon remains unclear, and it needs to be further discussed and studied.

Neuroimaging studies have shown that there are abnormalities in brain function and structural networks in AD and patients with a pre-existing condition, especially in the Default Mode Network (DMN) and the Executive Control Network (ECN) (Dennis and Thompson, 2014; Zhu et al., 2016; Badhwar et al., 2017; Liu et al., 2018). We therefore used ACOEC-FD on the two networks to further explore the difference between AD patients and HC subjects. We chose nine regions of interest (ROIs) from the two networks based on the literature (Power et al., 2011). Detailed information is provided in Table 9.

**Table 9:** The ROIs of DMN and ECN.

**Net**	**ROI**	**Detailed description**	**Location**
DMN	(1)PCC	Posterior Cingulate Cortex	–9, –54, 24
	(2)LHIP	Left Hippocampus	–26, –20, –10
	(3)RHIP	Right Hippocampus	28, –19, –10
	(4)MPFC	Medial Prefrontal Cortex	48, –57, 24
ECN	(5)LDLPFC	Left Dorsolateral Prefrontal Cortex	–48, 27, 21
	(6)RDLPFC	Right Dorsolateral Prefrontal Cortex	54, 33, 24
	(7)LPPC	Left Posterior Parietal Cortex	–30, –69, 39
	(8)RPPC	Right Posterior Parietal Cortex	36, –66, 48
	(9)APFC	Anterior Prefrontal Cortex	–6, 33, 45

**Figure 9:**
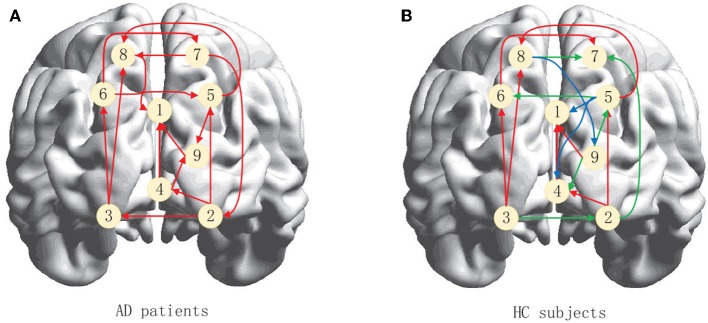
Brain effective connectivity networks learned by ACOEC-FD. **(A,B)** are graphical illustrations of the brain effective connectivity networks for AD patients and HC subjects, respectively. The red arrows in **(B)** indicate that the effective connectivity is the same as **(A)**, whereas blue arrows show additional connections, while green arrows show reversed connections.

Figure 9 summarizes the brain effective connectivity networks learned by ACOEC-FD. Compared with the HC subjects' EC network (Figure 9B), the AD patients' EC network (Figure 9A) has fewer connections. We also find that there are some opposing directions in AD patients between the left and right brain compared to HC subjects. This finding seems interesting as the neural influence and information transfer between the left and right brain may differ between HC and AD groups. In the current study, the reduction of EC has been confirmed to be closely related to AD disease, while the situation of reverse connection is rarely reported. However, the reverse of the brain EC may also be a cause of illness, which cannot be confirmed through common brain connectivity network research. Therefore, the ACOEC-FD algorithm can provide a new perspective for the diagnosis and analysis of AD disease.

In this study, our research was focused on AD patients, but actually the new method can also be applied to other brain diseases, i.e., epilepsy, schizophrenia, and autism, etc. Our future work will aim at applying the proposed methodology to other brain diseases.

## 5. Conclusions

By allowing stronger structural connectivity to lead to a greater probability of non-zero functional or effective connectivity, structural information has been incorporated into some studies to identify FC and EC. This paper proposes a new method, called ACOEC-FD, to learn EC from fMRI and DTI data. It uses DTI data to acquire anatomical constraint information, and constrains the search space of EC estimations. Then, by merging anatomical constraint information into the heuristic function, ACOEC-FD can select those ideal structures with stronger AC evidence. We used the generated data and real fMRI-DTI datasets to test our new algorithm in this study, and our experiments show some promising results and interesting observations. In general, our work contributes a novel EC learning framework of ACO merged DTI and fMRI data, which may have significant application potentials in cognitive neuroscience in the future.

## Data Availability Statement

Publicly available datasets were analyzed in this study. This data can be found here: https://ida.loni.usc.edu/login.jsp?project=ADNI&page=HOME.

## Ethics Statement

The studies involving human participants were reviewed and approved by ADNI. Written informed consent for participation was not required for this study in accordance with the national legislation and the institutional requirements.

## Author Contributions

JJ conceived the work. JL and AZo performed the experimental analysis. JJ and JL prepared the manuscript with revisions by AZh. All authors read and approved the final manuscript.

### Conflict of Interest

The authors declare that the research was conducted in the absence of any commercial or financial relationships that could be construed as a potential conflict of interest.
